# Robotic-assisted pulmonary lobectomy with lung cancer in a patient with situs inversus totalis

**DOI:** 10.1186/s13019-022-01983-8

**Published:** 2022-09-01

**Authors:** Chen Yang, Wenjian Jin, Xiao Fan, Liang Zheng, Hui Wang, Qianyun Wang

**Affiliations:** 1 Department of Thoracic Surgery, The First People’s Hospital of Changzhou, The Third Affiliated Hospital of Soochow University, Changzhou, 213003 Jiangsu China; 2 Department of Hepatobiliary Surgery, The First People’s Hospital of Changzhou, The Third Affiliated Hospital of Soochow University, Changzhou, 213003 Jiangsu China; 3 Department of Pathology, The First People’s Hospital of Changzhou, The Third Affiliated Hospital of Soochow University, Changzhou, 213003 Jiangsu China

**Keywords:** Situs inversus totalis, Lung cancer, Robot-assisted surgery, Lobectomy

## Abstract

**Background:**

Situs inversus totalis (SIT) is a relatively rare congenital abnormality in which the major thoracic and abdominal visceral organs are reversed from their usual positions. In patients with SIT and bronchial carcinoma, surgical difficulty increases sharply. It has been reported that the video-assisted thoracic surgery (VATS) still poses the operator to a challenge situation. The similarity of surgical positions and the flexibility of the mechanical arm in robotic surgery, may be beneficial to SIT patients due to reducing technical difficulties. Here, we present a first case of SIT patient with lung cancer, in which Da Vinci robot-assisted thoracic surgery (RATS) was performed successfully.

**Case presentation:**

A 66-year old patient, previously diagnosed with SIT since childhood, came to our hospital with two pulmonary nodules in his left lung field. The bigger one had increased somewhat for the last 2 years of follow-up. Software Mimics was preoperatively carried out to analyze anatomical variations. RATS was conducted to complete left upper lobectomy and left middle wedge resection. The patient had no intraoperative complications and was discharged day 5 after the operation.

**Conclusions:**

This is the first report of a successful robot-assisted lung cancer resection in a patient with SIT. In such challenging cases as lung cancer and rare anomaly as SIT, RATS is more advantageous and suitable than VATS with the help of software Mimics utilized for 3D reconstruction, which can identify the anatomical abnormalities and facilitate the surgical procedures.

## Introduction

Situs inversus totalis (SIT) is a rare anatomical congenital deviation characterized by a symmetrical and complete reverse anatomy of the major visceral organs within the thorax and abdomen. Fabricus in 1600 first reported and Sherk et al. [1] in 1922 first named this abnormality as SIT, which affects 1 individuals per 4000–8000 [1]. And the condition is typically incidentally revealed by radiographic examination [2] because it is often asymptomatic [3]. There is no definite correlation between SIT and the high risk of malignancy [4]. Many reports have revealed the association between SIT and various malignant tumors, including gastric cancer [5], rectal cancer [6], pancreatic cancer [7] and so on, but SIT with lung cancer is very rare. Several cases of video-assisted thoracic surgery (VATS) for lung cancer with SIT have been reported [8]. During the last decade, VATS has been developed as alternatives to thoracotomy for the majority of lung cancer patients. Although this minimally invasive surgical procedure was performed safely and feasibly, VATS for SIT is technically demanding because of the mirror image of the thoracic view, and improved surgical techniques are needed. Recently, robotic assisted lobectomy is a novel procedure for patients with pulmonary carcinoma and may bring good surgical outcomes [9]. The da Vinci Surgical Robot was developed to overcome the disadvantages of conventional VATS, which may include limited 2-dimensional imaging, limited dexterity of instruments and possible misalignment within surgeon’ hands. On the contrary, unique features of RATS, such as 3D view [10], precise moving multi-joint forceps with various degrees of freedom [11], and freedom from the constraints of the operator's position [12], are more suitable for patients with SIT.

However, a lot of surgeons can still be deceived by some technical traps during the surgery with SIT patients, especially confused with the pulmonary artery and pulmonary vein of lung segments [13]. Hence, different 3D imaging reconstruction software have been developed as powerful tools for the surgeons to gain stereoscopic images, detect anatomical structure, ascertain the exact location of the lung nodule and its relationship to blood vessels and bronchi, and design a perfect preoperative surgical planning. We present this case in which the Mimics Medical 21.0 software (Materialise, Belgium) [14] was used to construct three-dimensional computed tomography bronchography and angiography (3D-CTBA), which assisted us to achieve a meticulous, personalized, safe and anatomical thoracoscopic lobectomy.

To the best of our knowledge, there have been no reports on RATS for lung cancer in patients with SIT. In this paper, we firstly describe a patient with SIT who underwent thoracic lobectomy using the da Vinci surgical system assisted by 3D-CTBA preoperatively, which could help reduce the risk of intraoperative injury.

## Case report

The patient was a 66-year-old man with complete situs inversus that was identified in early youth. In October 2021, the man consulted our outpatient clinic for two pulmonary nodules discovered by computed tomography (CT) 2 years ago during his routine health checkup. The patient was referred to our hospital for detailed examination and surgical treatment because his bigger pulmonary nodule was developed and increased significantly.

**Fig. 1:**
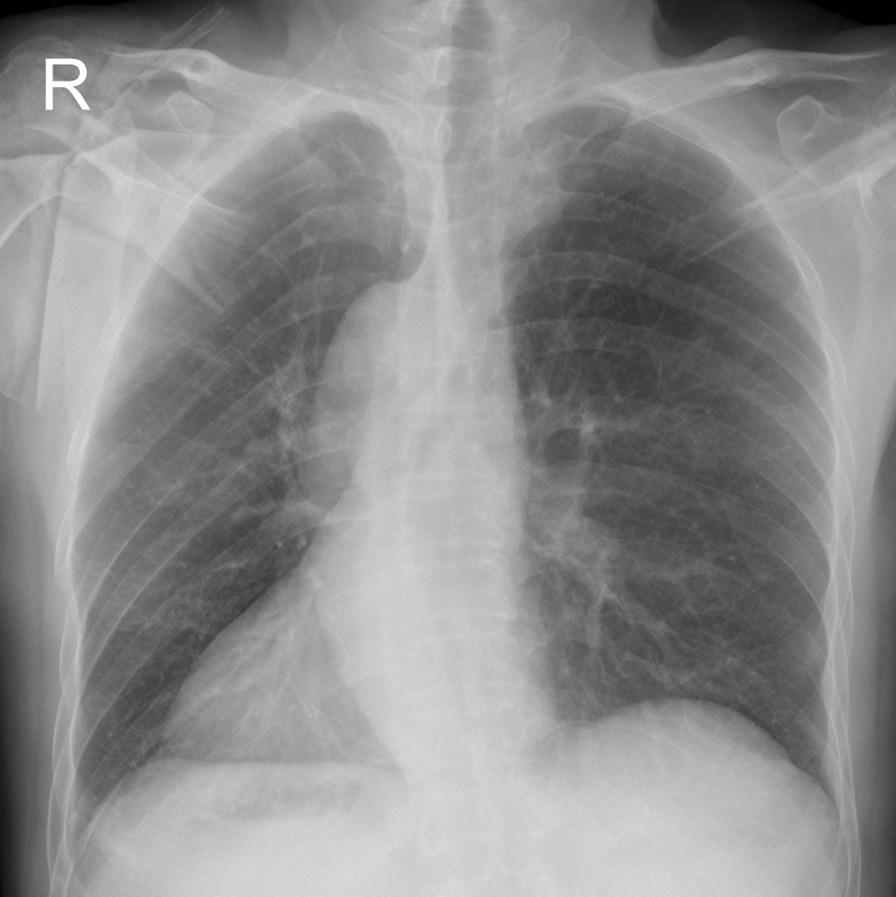
Chest radiograph on admission shows dextrocardia and positioning of the aortic arch on the right side, no tumor shadow could be seen

**Fig. 2:**
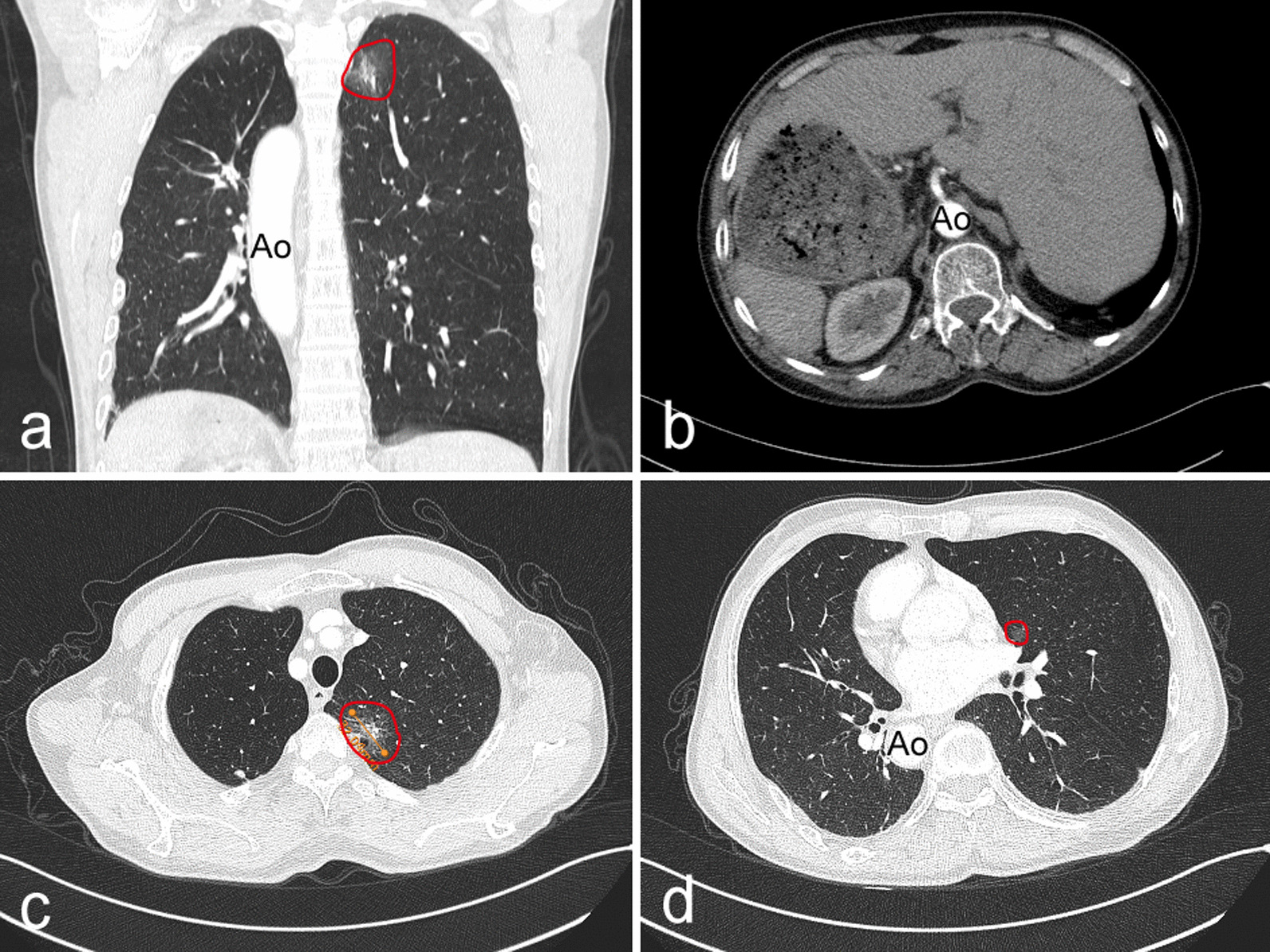
Computed tomography (CT) images. **a**: a mixed ground-glass opacity (mGGO) (measuring 34 × 32 mm in diameter) in the left upper lobe and mirror image of the aotic artery in the coronal section. **b**: abdominal organs presented with complete situs inversus. **c**: mGGO in the left upper lobe in the sagittal section. **d**: a pure ground-glass opacity (pGGO) measuring 0.8 cm in diameter closer to the mediastinum in the medial segment of left middle lobe

The patient had no recent history, such as cough, fever, chest pain and blood sputum. Also he had no previous medical history, whereas his smoking history lasts about 30 years, 90-pack/year. His grandmother once died of lung cancer while his mother died of breast cancer. Upon admission, vital signs were normal. For example, body temperature was 37℃, heart rate was 85 beats/min, respiration rate was 12 breaths/min, and blood pressure was 128/85 mmHg. Hematological tests, urinalysis and stool analysis revealed no abnormal findings. Physical examination was done methodically which revealed that the apex beat was located in a mirror image of those normally found on the left side. All serum tumor markers were within their normal ranges except carcinoembryonic antigen (CEA), which was 5.5 ng/mL. In addition, respiratory function, arterial gas analysis, electrocardiogram and bone scan were performed to rule out surgical contraindications, which showed no abnormalities. Chest X-ray revealed dextrocardia (Fig. 1) and right aortic arch, but no tumor shadow could be differentiated. Contrast enhanced CT scans of the brain, the chest and abdomen showed typical radiographic features of SIT (Fig. 2) and two GGO located in the left upper lobe and middle lobe separately with no swollen mediastinum and hilar lymph nodes and/or distant metastases (Fig. 2c, d). The two nodules were presented as ground-glass opacity (GGO), in which the bigger one was a mixed GGO adjacent to the mediastinal pleura, 3 × 4 cm in diameter, in the apical segment of left upper lobe (Fig. 2c) and the smaller one was a pure GGO closer to the mediastinum, 0.8 cm in diameter, in the medial segment of left middle lobe (Fig. 2d). Bronchoscopic examination showed that the arrangement of the bronchi were mirror images of those normally found on the other side, and that tracheal and bronchial mucosa were normal. Trans-bronchial tumor biopsy and positron emission tomography–computed tomography (PET/CT) was not performed because the tumor was not a solid tumor and could not be identified by chest radiography.

**Fig. 3:**
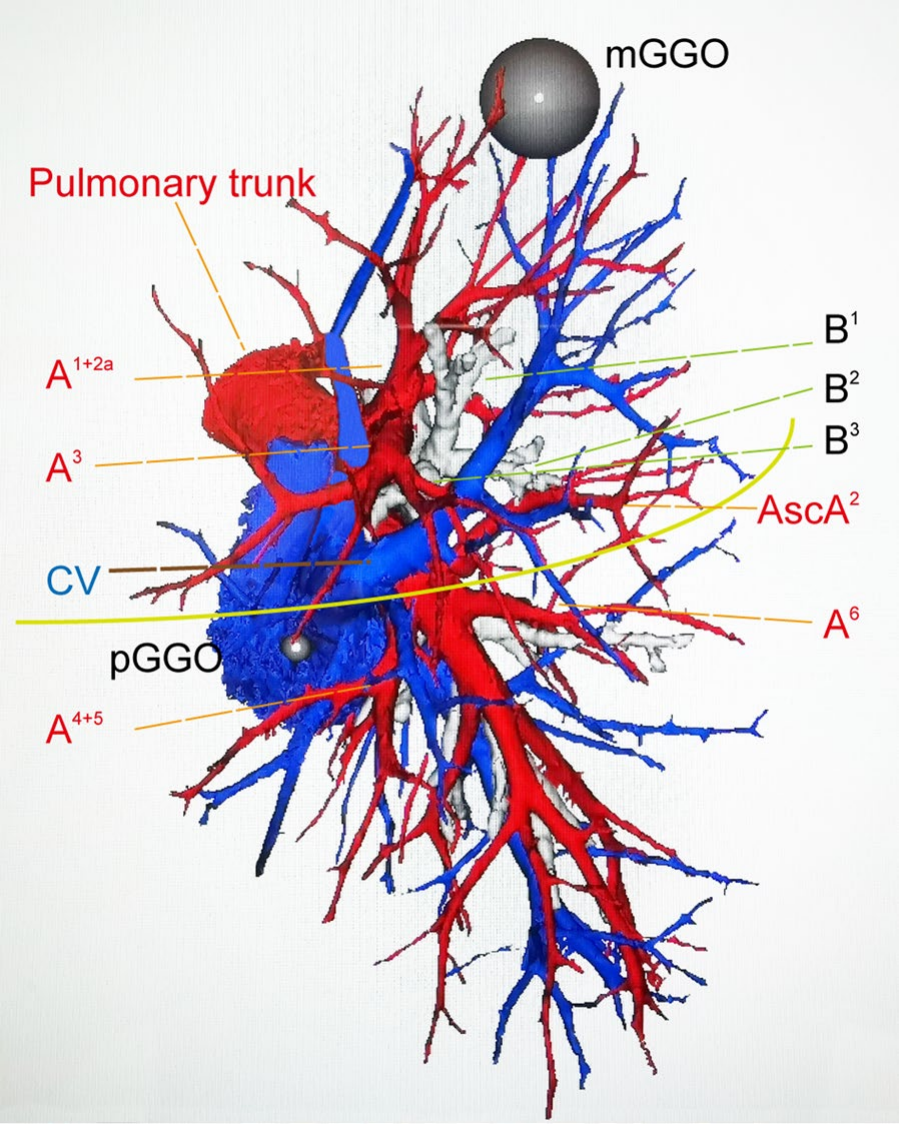
3D images reconstructed with Mimics Mecdical 21.0 software. Exact three-dimensional relationships between pulmonary anatomical structures and two ground-glass opacities (mGGO and pGGO); Grey: the GGO lesion; Blue: pulmonary veins; Red: pulmonary artery; White: bronchus. The yellow curve denotes the left upper lobe margin. AscA: Ascending artery

The patient used thin-Sect. (1 mm), enhanced CT (manufactured by American GE) scanning of the chest to obtain the images, which were recorded as digital imaging and communications in medicine (DICOM) data and subsequently transmitted to a computer and reconstructed with the Mimics Medical 21.0 software installed on a Windows 10 platform (manufactured by Huawei Technologies Co., Ltd). According to instructions of the software, three-dimensional computed tomography bronchography and angiography (3D-CTBA) was reconstructed to separate pulmonary structures from each other, including GGO, bronchi, pulmonary arteries, and veins, which were marked out in different colors (Fig. 3). The result of reconstruction was consistent with the diagnosis of SIT, one GGO in the upper left lung and the other nodule in the middle left lung. We could rotate the three-dimensional model arbitrarily on the computer, so as to further analyze the targeted structure carefully, identify the vascular variations except SIT and ascertain the surgical margin, which finally helped us facilitate the discussion of the surgical procedure and make reasonable preoperative decisions.

**Fig. 4:**
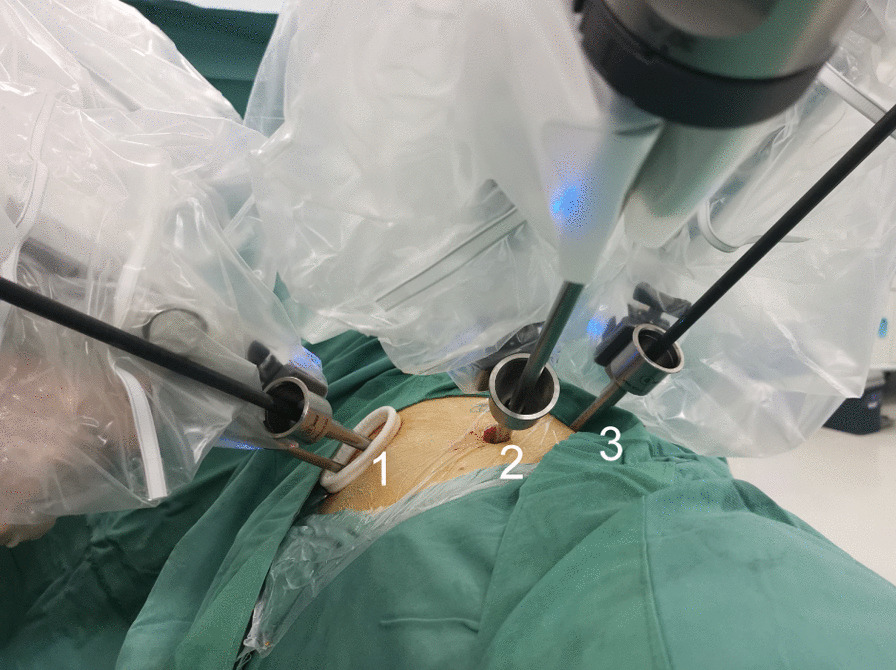
Patient positioning and port placement of RATS. Port 1: the sixth intercostal space of anterior axillary line, arm 1 and assistant hole; Port 2: the eighth intercostal space of the midaxillary line, camera port; Port 3: the eighth intercostal space of the rear axillary line, arm 2

**Fig. 5:**
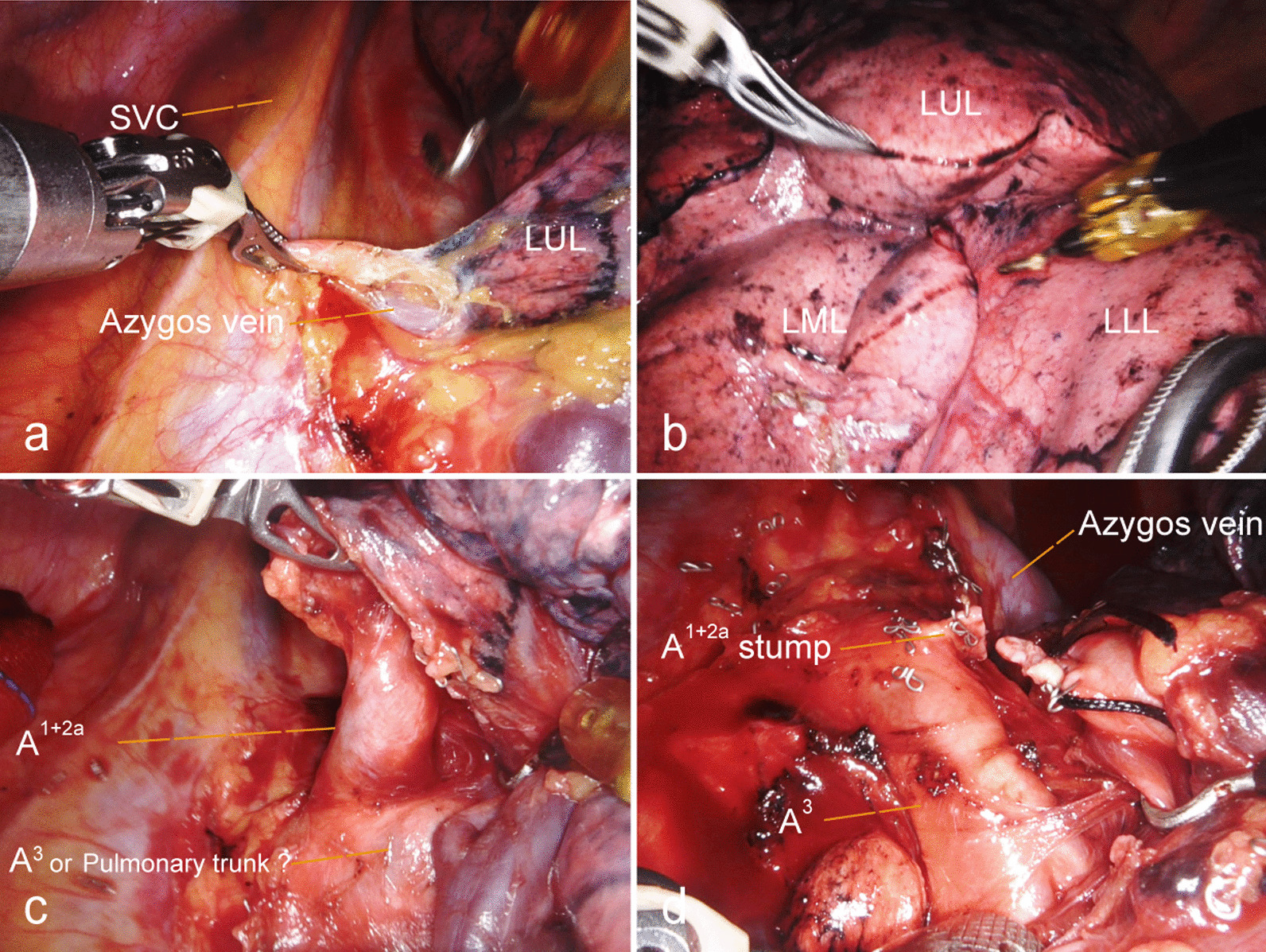
Intraoperative views. **a**: The location of the superior vena cava and azygos vein was a mirror image of the normal location and the aortic arch was missed in the left thoracic cavity; **b**: 3 lobes (LUL, LML, LLL) were separated by poor-defined fissures; **c**: Anatomical variation of the left upper lobe pulmonary arteries, confusion of A^3^ and pulmonary trunk; **d**: A^3^ was confirmed

Because the possibility of variations of the pulmonary vessels and the abnormality of SIT were taken into consideration, the operation was performed via robot-assisted thoracic lobectomy. The surgical procedures were as follows. The patient was administered general anesthesia with single-lung ventilation, which was performed with double-lumen endotracheal tube. And he lied on his healthy side in a lateral position and expanded his intercostal space. The robot was placed behind the patient’s head. The port placement is shown in Fig. 4 as what we done in our usual robot-assisted left lung surgery. For the da Vinci Xi system, we use three ports: a 8-mm camera port incised in the eighth intercostal space of the mid axillary line, a 3 cm operation port was set at the sixth intercostal anterior axillary line for the operating hole before the 30-degree 3D endoscope was inserted into the thorax. The incision was made for the assistant to staple and exchange the items of rolled-up sponges, titanium clips and hem-o-lok clips, as well as the left robotic arm working channel after placing the trocar sleeve. The last 8-mm port used for the right operated robotic arm was located in the eighth intercostal space of the rear axillary line. Slight changes to these locations are necessary once the dextrocardial anatomy is visualized, for example, the location of the observation port was less lateral than usual with the heart absent from our surgical area in this SIT patient. During surgery, the superior vena cava and azygos vein were located in the mirror images of their normal distributions, the aortic arch disappeared from the left thoracic cavity (Fig. 5a), which was relatively easy for mediastinal lymph node dissection. The left lung was composed of 3 lobes separated by poor-defined fissures (Fig. 5b). With the poor-developed pulmonary fissures, we designed to deal with the blood vessels from the pulmonary hilum at the beginning of the surgery. When we incised the mediastinal pleura above the posterior hilum of the upper lung, apical branch of pulmonary artery (A^1^)was exposed, as well as thick anterior branch of pulmonary artery (A^3^), which was confused with the superior trunk of the pulmonary artery (Fig. 5c). Although careful identification of the targeted blood vessels could be gained from preoperative 3D-CTBA imaging, we just cut off A^1^ and A^2a^ without A^3^ to avoid accidental injury of blood vessels. Afterwards, with the help of precise moving multi-joint forceps of da Vinci, the assistant lifts the left upper lobe (LUL) up with oval forceps while the arm 1 and the arm 3 were used to dissect the oblique fissure between the LUL and the left lower lobe (LLL) with a stapler, which was called the tunnel technique [15]. As this dissection proceeds, the pulmonary artery branches to the lingula become visible in the fissure, including posterior ascending branch artery (A^2^), A^3^, central vein (CV) and pulmonary trunk. The smaller branch artery (A^2^) was directly resected using an ultrasonic knife, while larger branch (A^3^) and left upper lobe bronchus was cut and stapled with a linear cutting stapler. Then the dissection of pulmonary veins and pulmonary bronchus were performed after we ensured the lowest branch of the upper pulmonary veins was the middle lobe vein, which represents the usual pattern of the right side. Finally, the horizontal fissure was cut and the specimen was stapled shut. The other smaller pure GGO was located in the left middle lobe near the middle lobe vein root adjacent to the mediastinal pleura according to the CT images and 3D-CTBA reconstruction. The lesion was resected after the lung surrounding the GGO was retracted posteriorly and isolated from the hilum and the middle vein. As the aortic arch was absent within the left thoracic cavity, systemic lymphadenectomy was relatively easy. The No. 10, 11, and 7 lymph nodes were dissected. The total operation time was two hours, with blood loss of 50 mL.

The final histopathology showed that the bigger nodule was highly differentiated invasive adenocarcinoma(3.8 cm in diameter), composed of a mixture of acinar subtype and adherent subtype, and the smaller nodule was adenocarcinoma in situ with a tumor diameter of 0.8 cm. Lymph nodes were pathologically negative. The postoperative course was uneventful, and the patient did not need chemotherapy or radiotherapy, only with the regular follow-up at the outpatient clinic. Six months after the operation, the patient is doing well with no signs of recurrence.

## Discussion

With the gradual improvements of people’s health consciousness and advances in imaging techniques, the increased popularity of health checkups, for example, low dose CT screening and high-resolution CT scanning, have brought out a rise of early detection rate of lung cancer each year [16]. Video-assisted thoracic surgery (VATS) for early lung cancer can maximally achieve minimally invasive treatment of patents and alleviate their postoperative pain, which has been developed as alternatives to thoracotomy with its benefits for improving quality of life and reducing complications. Since Morgan [17] in 2003 firstly reported robotic lobectomy, the use of Da Vinci robot-assisted thoracic surgery (RATS) has become increasingly common. RATS is the key surgical approach for experienced surgeons in some situations where the airway needs to be reconstructed, locally invasive T4 or N2 lesions pre-treated with neoadjuvant therapy require radical resection [18]. Some researchers have pointed that the operating feeling of the surgeon during RATS was more likely as thoracotomy surgery [19], in addition to the advantages of 3D image view, automatic filtering function and flexible internal wrist rotation system [20]. At the same time, there is no need for the operator to consider the standing position and the inadaptation of using devices by non-dominant hand in various situations, he can freely approach either left or right side with the mechanical arms [21]. So RATS is considered to be suitable for the situation in which the left and right organs are reversed from normal position as in SIT.

Notably, compared to the normal anatomy, the anatomical structure of the SIT is relatively complex and prone to vascular variation, thus the risks and challenges during the SIT patients’ surgery are increased. There have been some reports of thoracoscopic lobectomy and segmentectomy for SIT cases [8, 22]. They have found that it was necessary to reverse the surgical procedures because of the complete transposition of the viscera [23], difficulties in confirming surgical anatomy and difficulty in the anatomical orientation hence causing confusion intraoperatively [24, 25]. While in this case with SIT, we construct a 3D image of the pulmonary structures assisted by the software Mimics Medical 21.0 and designed a sound surgical plan preoperatively to avoid accidental damage. We planned to perform left upper lobectomy with single-direction method [26], which was routinely used in segmentectomy and lobectomy with incomplete interlobar fissures. The dissecting order followed by the A^1^, A^3^, upper lobe vein, A^2^, bronchus and the fissure due to the fused oblique fissure and horizontal fissure. As A^1^ was resected, the thick pulmonary artery below the root of A^1^ was thought to be A^3^ or the superior trunk of the pulmonary artery. Even though this artery was more likely to be A^3^ on the basis of the anatomical relations in 3D-CTBA image, the dissection was not performed immediately. Subsequently, we dissected the oblique fissure from the pulmonary hilum in turn. As can be seen from Fig. 5d, just this surgical process demonstrated the advantages of RATS compared to VATS extremely. With the help of freely precise moving multi-joint forceps, we used the fissure tunneling technique [15] to identificate the bronchial and vascular structures correctly and reduced the risk of air leakage. Once the fissure was completed, it was more feasible to obtain the view of the broncho-vascular anatomy. Consequently, A^3^ was easily confirmed and was divided with a linear cutting stapler. As we all know, during RATS, robotic arms and tools made systemic lymph node dissection more precise.

Regarding this surgical procedure in SIT patients with lung carcinoma, although previous reports showed cases of segmentectomy, lobectomy or pneumonectomy with the help of VATS, we did not find any case with the use of RATS in our literature review. Although Baruah [27] first reported the case of lung cancer with SIT in 1952 and Kodama performed the first lung cancer surgery in SIT patient in 1990 [28], surgery qualification for lung cancer/SIT patients is still difficult as there is a little experience of less than 30 lung cancer/SIT patients operation so far, among which only eleven cases were performed with video-assisted thoracic surgery [1, 13, 29–37]. The majority of the study group was male (8/11), and adenocarcinoma was the most frequent pathological type (7/11). Recent researchers reported that the patient with SIT has more frequent major vessel abnormalities compared to the general population so that they recommended 3D reconstruction (Fig. 3) to avoid iatrogenic injuries and catastrophic bleeding [1, 8, 13].

It is the first time we have performed robotic-assisted pulmonary lobectomy with lung cancer in a patient with SIT safely and successfully. Though we complete the operation eventually, SIT still is a challenge to surgeons, especially when meeting various abnormal anatomical structures and poor developed fissures, which can benefit the advantages of 3D reconstruction and Da Vinci robot.

## Conclusion

RATS is a novel revolutionary surgical platform, whose unique advantages made it more suitable for SIT patients with lung cancer and help surgeons be at ease and safe when performing such challenging procedures.

## Data Availability

The datasets used are available from the corresponding author on reasonable request.

## Abbreviations

SIT: Situs inversus totalis
VATS: Video-assisted thoracic surgery
RATS: Robot-assisted thoracic surgery
LUL: Left upper lobe
LML: Left middle lobe
LLL: Left lower lobe
